# Expression of Cancer/Testis Antigens is Correlated with Improved Survival in Glioblastoma

**DOI:** 10.18632/oncotarget.950

**Published:** 2013-04-15

**Authors:** Marcelo Roberto Pereira Freitas, Suzana Maria Fleury Malheiros, João Norberto Stávale, Thais Priscila Biassi, Fernando Tadeu Zamunér, Maria Dirlei Ferreira de Souza Begnami, Fernando Augusto Soares, Andre Luíz Vettore

**Affiliations:** ^1^ Cancer Molecular Biology Laboratory, Department of Science Biology, Federal University of São Paulo, Rua Pedro de Toledo, São Paulo, SP, Brazil; ^2^ Department of Neurology, Federal University of São Paulo, Rua Botucatú, São Paulo, SP – Brazil; ^3^ Department of Pathology, Federal University of São Paulo, Rua Botucatú, São Paulo, SP – Brazil; ^4^ Department of Pathology, A C Camargo Cancer Hospital, Rua Prof. Antônio Prudente, São Paulo, SP, Brazil

**Keywords:** Brain cancer, Glioblastoma, GBM, Cancer/Testis antigens, CTA expression

## Abstract

**Background:**

Glioblastoma (GBM) confers a dismal prognosis despite advances in current therapy. Cancer-testis antigens (CTA) comprise families of tumor-associated antigens that are immunogenic in different cancers. The aim of this study was to determine the expression profile of a large number of CTA genes in GBM.

**Methods:**

We selected, from 153 CTA genes, those genes potentially expressed in GBM. The expression pattern of 30 CTA was then evaluated by RT-PCR in a series of 48 GBM and 5 normal brain samples. The presence of CTCFL protein was also evaluated by immunohistochemical staining.

**Results:**

Among the genes with no expression in normal brain, ACTL8 (57%), OIP5 (54%), XAGE3 (44%) and CTCFL (15%) were frequently expressed in GBM, while over 85% of the tumors expressed at least 1 of these four CTA. Coexpression of two or more CTA occurred in 49% of cases. CTCFL protein expression was detected in 13% of the GBM and was negative in normal brain samples. GBM expressing 3-4 CTA was associated with significantly better overall survival (OS) rates (P = 0.017). By multivariate analysis, mRNA positivity for 3-4 CTA (P = 0.044), radiotherapy (P = 0.010) and chemotherapy (P = 0.001) were independent prognostic factors for OS.

**Conclusions:**

GBM frequently express ACTL8, OIP5, XAGE3 and CTCFL. A relatively high percentage of tumors expressed at least one of these four CTA, opening the perspective for their utility in antigen-specific immunotherapy. Furthermore, mRNA positivity for 3-4 CTA is an independent predictor of better OS for GBM patients.

## INTRODUCTION

Glioblastoma is the most common primary malignant brain tumor in adults. The current treatment of GBM, which consists of surgical resection followed by radiation therapy and chemotherapy, has a median survival of 14.6 months (1). Moreover, conventional therapy is often associated with considerable local and systemic side effects. Therefore, the development of novel therapeutic approaches is essential to improve the outcome of GBM patients (2, 3).

The discovery of tumor antigens has opened new doors for specific tumor-targeted treatments using passive or active immunotherapeutic strategies. One group of these antigens, the so-called cancer/testis antigens, is present in germ cells in a variety of cancers yet rarely found in normal adult tissues (4-6). The present catalog of CTA comprises more than 200 distinct genes (7). As spontaneous anti-tumor immune responses can be elicited by CTA, they represent promising candidates for cancer immunotherapy. In recent years, several CTA, particularly MAGE-A3 and NY-ESO-1, have been studied as target antigens in vaccine clinical trials for many cancer types (8), including GBM (9).

The expression frequencies of some CTA in GBM have been determined but little is known about their composite expression (10-15). This information is crucial in designing immunotherapeutic strategies, because it is mandatory to know what proportion of patients can be expected to be eligible and which antigens have to be chosen (16). To better understand the expression profile of CT antigens in GBM, we conducted an extensive expression analysis of these genes in glioblastoma and normal brain specimens from adult patients.

## RESULTS

### Patient baseline characteristics

Forty-eight patients with histologically diagnosed glioblastoma (WHO Grade IV), that had fresh frozen samples available, were enrolled in this study (Supplementary Table S2). One patient was treated in another hospital and was lost to follow-up. The sample comprised 14 (29%) women and 34 (71%) men, with a median age of 54 (±14) years. Twenty-one patients (45%) had a Karnofsky performance status (KPS) >70. All patients underwent biopsy (23%) or resection of the tumor (44% subtotal and 33% gross total resection), and 64% of patients received standard, external beam radiation therapy. At least 43% of patients received adjuvant chemotherapy.

### Selection of CTA genes

Candidate CTA genes with a high probability of being expressed in GBM were selected by *in silico* screening using all 153 CTA genes previously described by Hofmann et al (5). First of all, 14 CTA genes classified as testis/brain restricted by Hofmann et al were excluded. The remaining 139 CTA genes were used to search the NCBI-CGAP SAGE database, Oncomine and NExtBio collections of microarray data, published data of CTA mRNA expression (CTDatabase) and high-throughput expression data also provided by Hofmann et al. Ultimately, a total of 30 CTA were selected (*ACTL8*, *BAGE, BRDT, CSAG2, CTAG1A, CT45, CTCFL, CXORF48, CXORF61, DDX53, FTHL17, MAGEA1, MAGEA2, MAGEB4, MAGEB6, NFX2, OIP5, PBK, ROPN1, SCLO6A1, SPANXN1, SPANXN2, SPANXN3, SPANXN4, SPINLW1, SPO11, SSX7, XAGE1, XAGE3* and *ZNF165*) for assessment of gene expression in a context of potential high expression in GBM samples and absence in normal brain.

### Analysis of CTA genes expression in GBM

Based on the above results, 30 CTA genes were selected for evaluation in GBM and normal adult tissue samples using RT-PCR analysis. Representative examples of RT-PCR results are shown in Supplementary Figure 1.

Due to the limited quantity of RNA of many samples and the number of genes selected, it would have been virtually impossible to evaluate all possible candidate-genes in all samples. Consequently, it was decided to first conduct a discovery study to yield a more limited set of “best” CTA genes for use in the validation set. The first step was to verify the expression status of 30 CTA genes in 17 GBM samples. According to this analysis, no expression of *BRDT, CSAG2, CTAG1A, CT45, CXORF61, MAGEA2, MAGEB4, SLCO6A1, SPANX N1, SPANX N2, SPANX N3, SPANX N4* or *SSX7* was detected in the GBM samples examined, whereas *MAGE-A1* (6%), *MAGE-B6* (6%), *BAGE* (12%), *SPO11* (12%) *NFX2* (18%), and *XAGE1* (18%) transcripts were rarely detected. On the other hand, 11 CTA genes, *CXORF48* (35%), *ROPN1* (82%), *DDX53* (94%), *ZNF165* (100%), *PBK* (100%), *FTHL17* (76%), *SPINLW1* (46%), *CTCFL* (25%), *XAGE3* (94%), *OIP5* (29%) and *ACTL8* (46%) were frequently expressed in GBM cases (Table 1).

**Table 1:** Expression of the 30 selected CTA genes in glioblastomas and normal brain controls

	GBM samples		Normal Brain (5)
CT Genes			% (n)
	Discovering Set (17)	Validation Set (48)	
	% (n)	% (n)	
			
BRDT	0 (0)	-	-
CSAG2	0 (0)	-	-
CTAG1A	0 (0)	-	-
CT45	0 (0)	-	-
CXORF61	0 (0)	-	-
MAGEA2	0 (0)	-	-
MAGEB4	0 (0)	-	-
SLCO6A1	0 (0)	-	-
SPANX N1	0 (0)	-	-
SPANX N2	0 (0)	-	-
SPANX N3	0 (0)	-	-
SPANX N4	0 (0)	-	-
SSX7	0 (0)	-	-
MAGEA1	6 (1)	-	-
MAGEB6	6 (1)	-	-
BAGE	12 (2)	-	-
SPO11	12 (2)	-	-
NFX2	18 (3)	-	-
XAGE1	18 (3)	-	-
CXORF48	35 (6)	-	100 (5)
SPINLW1	46 (8)	-	60 (3)
FTHL17	76 (13)	-	80 (4)
ROPN1	82 (14)	-	100 (5)
DDX53	94 (16)	-	100 (5)
ZNF165	100 (17)	-	100 (5)
PBK	100 (17)	-	100 (5)
CTCFL*	24 (4)	15 (7)	0 (0)
OIP5	29 (5)	54 (26)	0 (0)
ACTL8*	46 (8)	57 (21)	0 (0)
XAGE3	94 (16)	48 (23)	0 (0)

*CTCFL and ACTL8 were analyzed in 46 and 37 GBM samples, respectively.

In the next step, the expression pattern of these 11 CTA genes in five normal brain specimens was evaluated using the same conditions defined for the tumor samples. This analysis showed that only *ACTL8, CTCFL, OIP5* and *XAGE3* were not expressed in normal brain. Hence, expression of these four antigens was evaluated in the validation set revealing that *ACTL8* had the highest incidence of mRNA positivity (57%), followed by *OIP5* (54%), *XAGE3* (48%) and *CTCFL* (15%) (Table 1). Notably, 86% of the GBM cases showed expression of at least one CTA gene from the panel selected. Among them, 39% of the cases expressed only one of these CTA genes. Coexpression of two, three and four CTA genes occurred in 25%, 17% and 5% of the examined GBM cases, respectively.

**Figure 1:**
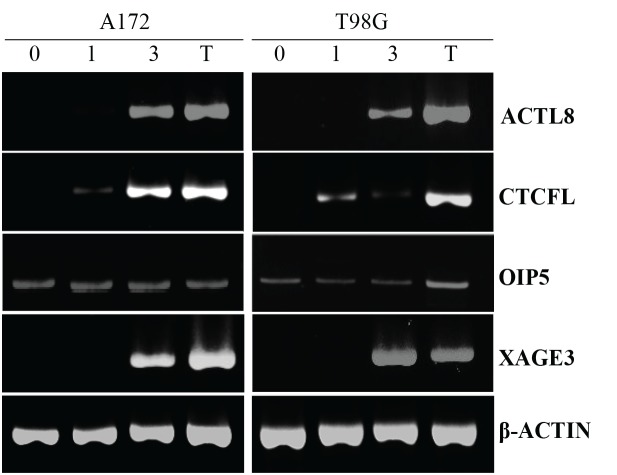
RT -PCR for *ACTL8*, *CTCFL*, *OIP5* and *XAGE3* expression in GBM cell lines treated with decitabine (DAC) A172 and T98G cell lines were treated with 5mM DAC for 3 days. (0) untreated; (1) one-day DAC treatment; (3) three-day DAC treatment; (T) testis tissue was used as a positive control. Amplification of β-ACTIN is shown as reference. Only *OIP5* was expressed in these cell lines before DAC treatment.

The expression of these four CTAs was also examined in two GBM cell lines (A172 and T98G) and only *OIP5* transcripts were detected in these cell lines (Figure 1).

### Expression of CTCFL, XAGE3, OIP5 and ACTL8 in Human Normal Tissues

**Figure 2:**
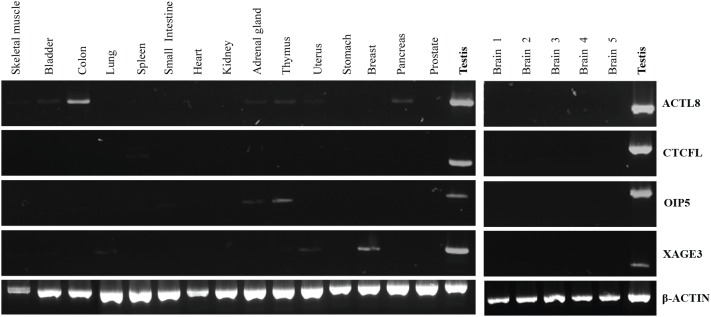
RT -PCR analysis of *ACTL8*, *CTCFL*, *OIP5* and *XAGE3* expression in normal tissues A commercially available panel of normal adult tissues (Clontech) was evaluated (left panel). The normal brain samples (right panel) were collected from patients with mesial temporal sclerosis during temporal lobectomy. An equal amount of testis cDNA was used as a representative positive control.

The expression of the four selected CTA genes was investigated by RT-PCR in a panel of 16 distinct normal tissues. As can be seen from Figure 2, all four CTA genes under investigation were not expressed in normal brain, but presented low expression at different frequencies in other normal adult tissues. *ACTL8* was expressed in normal colon, skeletal muscle, bladder, adrenal gland, thymus, uterus and pancreas, whereas *CTCFL* expression was detected only in spleen. *OIP5* was expressed in thymus, adrenal gland and small intestine while *XAGE3* transcripts were present in skeletal muscle, bladder, lung, thymus, uterus and breast. The expression of these CTA genes was not detectable in other normal tissues evaluated.

### Immunohistochemical analysis of CTCFL protein

**Figure 3:**
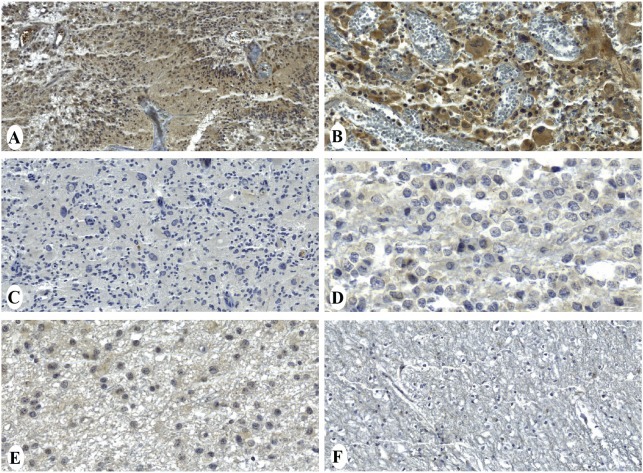
Immunohistochemical staining of *CTCFL* protein in gliomas and normal brain tissue GBM samples showing diffuse cytoplasmic staining (A, B) and negative expression (C). Anaplastic astrocytoma (D), diffuse astrocytoma (E) and normal brain tissue (F). Magnification: 50μm (B, D, F) and 100μm (A, C, E).

The RT-PCR technique is a sensitive method for the detection of CTA messenger RNA synthesis, but in order to verify that CTA genes are also expressed at the protein level, sectioned materials from formalin-fixed GBM (92), anaplastic astrocytomas (15), grade II astrocytomas (48) and grade I astrocytomas (38) were analyzed by IHC. Protein expression of CTCFL was the only analysis performed because antibodies against XAGE3, OIP5 and ACTL8 for immunohistochemical assays were unavailable. Diffuse cytoplasmic staining for CTCFL was present in 13% (12/92) of GBM cases (Figure 3). Immunoreaction to *CTCFL* was negative in all anaplastic astrocytomas (0/15), diffuse astrocytomas (0/48), pilocytic astrocytomas (0/38) and normal brain samples (0/40) examined.

### Up-regulation of CTA expression on GBM cells by DAC treatment

To determine whether the treatment of GBM cell lines with DAC would alter the expression of *ACTL8, CTCFL, OIP5* and *XAGE3*, two GBM cell lines were treated with 5μM DAC, the mRNA extracted and RT-PCR performed. Data presented in Figure 1 demonstrates that T98G and A172 cells did not express *ACTL8, CTCFL* and *XAGE3*, yet after three days of exposure to DAC, these cell lines presented a remarkable expression of these antigens. Both cell lines expressed *OIP5* and DAC treatment did not affect its expression. β-ACTIN (an endogenous control) was equally expressed in all cell lines before and after DAC treatment

### Prognostic value of CTA expression in GBM patients

Significant associations between clinical characteristics of GBM patients (age, KPS, extent of resection, radiotherapy and chemotherapy) and expression of *ACTL8, CTCFL, OIP5* and *XAGE3*, as well as, coexpression of 3-4 CTAs, were evaluated. Nevertheless, no significant correlation was observed between the expression of these CTA genes and any of the clinical parameters analyzed (Supplementary Table S3).

**Figure 4:**
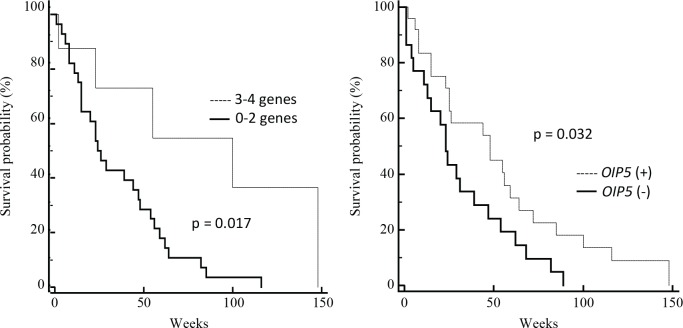
Correlation between CTA gene expression and overall survival of GBM patients Kaplan-Meier survival estimates of patients were performed according to RT-PCR positivity of OIP5 and coexpression of multiple CTA (0-2 versus 3-4).

Additionally, we investigated the correlation of the mRNA expression of the four selected CTA genes, individually or as a number of coexpressed genes, with overall survival. The *OIP5*-positive patients had a longer survival period than the *OIP5*-negative group (48 weeks *versus* 23 weeks) and this difference was statistically significant (P = 0.032, hazards ratio = 0.53, 95% CI = 0.28-1.0; Figure 4). Further, patients whose tumors coexpressed 3-4 CTA genes also showed better overall survival (100 weeks 3-4 CTA *versus* 26 weeks 0-2 CTA, P = 0.017, hazards ratio = 0.36, 95% CI = 0.17-0.74; Figure 4). RT-PCR positivity for *ACTL8, CTCFL* and *XAGE3* was not associated with survival outcome in this cohort. It is noteworthy that an association between age and survival was also found as expected in a GBM population (data not shown).

To detect independent predictors of survival, an analysis of prognostic variables based on the Cox proportional hazards model was performed involving significant clinical features (age, KPS, extent of resection, radiotherapy and chemotherapy) and molecular variables (*OIP5* expression and coexpression of 3-4 CTA genes) associated with survival probability. This multivariate survival analysis revealed that coexpression of 3-4 CTA genes (P = 0.044, hazards ratio = 0.3, 95% CI = 0.093-0.963), radiotherapy (P = 0.010, hazards ratio = 0.16, 95% CI = 0.04-0.65) and chemotherapy (P = 0.001, hazards ratio = 0.10, 95% CI = 0.03-0.41) remained as independent predictors of overall survival (Table 2).

**Table 2:** Multivariate analysis of overall survival (Cox regression model)

Variables	Hazards ratio	95% CI	p
Radiotherapy	0.16	0.04-0.65	0.010
Chemotherapy	0.10	0.03-0.41	0.001
3-4 CTA genes positivity	0.30	0.093–0.963	0.044

## DISCUSSION

Glioblastoma is one of the most lethal human cancers, with very few long-term survivors (1). Despite aggressive multimodal therapy, including surgery, radiation and chemotherapy, the prognosis for patients with this high-grade astrocytoma remains poor. Thus, glioblastoma is a relevant clinical problem, while successful strategies for its treatment have remained elusive.

Cancer vaccines are a promising cancer treatment due to low toxicity, the potential for circumventing drug cross-resistance and the potential for persistence of the antitumor effect caused by immunologic memory (17). There is a growing interest in applying tumor immunotherapy approaches to primary brain tumors, boosted by the recent FDA approvals of Sipuleucel-T for prostate cancer and Ipilimumab for metastatic melanoma (18, 19). In order to search for new tumor-associated antigens capable of inducing a tumor-directed immune response and to contribute to the development of immunotherapy approaches for glioblastomas, the present study evaluated a broad panel of CTA genes in a large number of GBM samples.

To date, few studies have attempted to assess the CTA expression pattern in GBM and the limited data available is conflicting. According to reports, *MAGEA1* expression could be detected in 0-38% of GBM cases evaluated in three studies, whereas *SSX2* mRNA positivity was present in 0-29% of tumors examined by three independent investigations and *MAGEA3* (0-22%) and *SSX4* (0-27%) gene products were detectable in different proportions of grade IV astrocytomas. Other CTAs reported as occasionally expressed in GBM include *SSX1, LAGE1, BAGE, CT7, CT10, MAGEA3-6, MAGE4* and *MAGE10 (10, 11, 13, 20, 21)*.

Consistent with these results, we found *MAGEA1* (6%) and *BAGE* (12%) to be rarely expressed in GBM samples. Additionally, we detected the presence of *SPINLW1, ROPN1, ZNF165* and *PBK* transcripts in normal brain and this data is in accordance to the CTDatabase where these CTA are described as testis-selective. Surprisingly, we also found expression of *CXORF48, DDX53* and *FTHL17* in the normal controls, although these CTA are classified as testis-restricted in the CTDatabase (7).

A four-gene panel (*ACTL8, CTCFL, OIP5* and *XAGE3*) of CTA specifically and frequently expressed in GBM was identified. According to this data, 86% of the GBM cases presented positivity for at least one member of the four-gene panel and 47% of tumors investigated expressed more than one of these CTA genes. Of note, these four CTA genes showed no expression in normal brain tissues, but their expression is not restricted to testis and could be detected in few other normal tissues (testis-selective). Among the 15 normal adult tissues tested in our study, *CTCFL* was found expressed only in spleen, while a very low expression of *XAGE3, OIP5* and *ACTL8* could be detected in other distinct normal adult tissues. According to Caballero and Chen (4), many CTA genes showed low-level mRNA expression in a limited number of somatic tissues; however, this low-level CTA expression has never been confirmed at the protein level by immunohistochemical analysis with anti-CTA antibodies, and whether such basal RNA expression translates to a biologically significant (6,22).

The present study confirms that a large proportion of glioblastomas present CTA expression, suggesting that *ACTL8, CTCFL, OIP5* and *XAGE3* may be eligible for future vaccines clinical trials targeting multiple antigens; however, the demonstration of immunogenicity in the human host is crucial for these CTA be considered as a potential cancer vaccine target. Of note, a multi-epitope-pulsed dendritic cell vaccine (including synthetic class I peptides from MAGE1) for patients with newly diagnosed glioblastoma was recently tested in a phase I trial (9).

The biological function of CTA genes remains poorly understood. *CTCFL*, also known as *BORIS*, is a transcriptional regulator; it directs epigenetic reprogramming at CTCF target sites in normal and tumor tissues (23, 24). Furthermore, it has been proposed as a mediator of the induction/derepression of other CTA genes (8, 24). OIP5 seems to be involved in chromatin reorganization during the cell cycle (25). Previous studies have demonstrated high expression of *OIP5* mRNA in gastric, colorectal, lung and esophageal carcinomas, where it contributes to the growth of cancer cells (25, 26). No information about biological function of *ACTL8* and *XAGE3* is available.

Interestingly, some studies have failed to observe a clear correlation between mRNA level and protein expression of tumor antigens (11, 27). Although we have analyzed different patient cohorts by RT-PCR and immunohistochemical staining, our data correspond to similar expression frequencies at mRNA and protein levels of CTCFL (15% and 13%, respectively). Also noteworthy, immunohistochemical staining revealed that CTCFL expression was frequently detected in GBM specimens but was completely negative in all less aggressive astrocytomas (Grade I, II or III) cases. This finding is consistent with the previous observation that CTA antigens are more often expressed in tumors of higher histological grade and later clinical stage (28, 29). For instance, MAGE-A1 expression has been found in 48% of metastatic melanoma versus 16% of primary melanoma (30) and NY-ESO-1 has been found to be expressed in 40% of grade 3 bladder tumors and 23% of grade 2 tumors, but in none of the grade 1 tumors (31).

As mentioned above, CTA are suitable targets for immunotherapy of human malignancies, and different clinical trials are ongoing. Nevertheless, the heterogeneous intratumor expression of CTA may hamper the effectiveness of CTA-directed vaccination through the emergence of CTA-negative neoplastic clones. Therefore, it would be important to induce antigen expression in CTA-negative tumors prior to immunization (32). Methylation of CpG islands in CTA gene promoters is the primary silencing mechanism in healthy somatic tissues. Activation of CTA gene expression in tumors is thought to result from demethylation of these sequences (33). Our results demonstrated that DAC treatment should induce a reduction in DNA methylation and, consequently, increases the expression of CTA genes in GBM cells. Different studies have shown that DAC can upregulate the expression of CTA antigens on tumor cells, while it has been observed that DAC cannot induce CTA expression in cell lines derived from normal tissues as fibroblasts, endothelial cells, myoblasts, keratinocytes, and melanocytes (33-35). Although it is possible that CpG islands in the promoter regions are so densely methylated in normal cells as to render DAC ineffective, the possibility that other mechanisms of transcriptional, and even post-transcriptional, control of gene expression might be at play is highly likely. Taken together, these results suggest a potential utility of combining DAC therapy with CTA immunotherapy approaches as an alternative treatment option for GBM patients.

The identification of human tumor antigens could be important for the identification of molecular markers useful for diagnosis or prognosis (36). Clinical predictors of prognosis for patients with GBM include patient age and performance status, while molecular markers that effectively predict response to therapy and survival outcomes are limited (37). Currently, promoter methylation of the DNA repair gene O6-methylguanine methyltransferase (MGMT) is recognized as a reliable indicator of favorable response to radiotherapy and chemotherapy (38). In light of these considerations, our study identified a significant association between coexpression of 3-4 CTA genes and overall survival in patients with GBM, suggesting its use as potential a new prognostic indicator.

Most of previous studies have reported an association of CTA expression with poor outcome in multiple myeloma, ovarian cancer, lung cancer, head and neck cancer and gastric cancer (39-43). On the other hand, Sharma et al. described a positive association between expression of CT10 and improved survival for patients with urothelial carcinoma (44). Additional prospective studies with independent cohorts of GBM patients are needed to confirm the positive correlation between CTA expression and better disease outcome, but, based in our results, we can speculate that the presence of these CTA in glioblastoma tissues may have elicited a spontaneous immune response and this could impact favorably the prognosis.

In conclusion, the present study, one of the largest evaluating CTA expression in GBM reported to date, found that *ACTL8, CTCFL, OIP5* and *XAGE3* expression was characteristic of the majority of GBM examined. The coexpression of 3-4 of these CTA showed a significant association with increased overall survival, suggesting their use as a new prognostic marker and as potential targets for immunotherapy approaches in GBM treatment.

## MATERIAL AND METHODS

### Patients and tissue samples

A total of 48 tumor samples were obtained from GBM lesions excised from patients operated at São Paulo Hospital/ Federal University of São Paulo between 2002 and 2010. The glioma diagnoses were confirmed by a neuropathologist and all cases were classified morphologically according to the World Health Organization (WHO) classification. None of the patients had received prior cytotoxic or radiation therapy. All samples were checked microscopically for the presence of neoplastic tissue and the absence of contaminating normal brain tissue. Four normal brain specimens were collected from patients with mesial temporal sclerosis during temporal lobectomy at Sao Paulo Hospital (Sao Paulo, SP, Brazil). All tissue samples were snap-frozen in liquid nitrogen within 30 minutes of resection and stored at -80°C until RNA extraction. One normal brain RNA sample acquired from Clontech was also analyzed in the study.

Paraffin-embedded tissue samples from 92 glioblastomas, 15 anaplastic astrocytomas, 48 diffuse astrocytomas, 38 pilocytic astrocytomas and 40 normal brain samples were obtained from the files of the Department of Pathology of A. C. Camargo Hospital (Sao Paulo, SP, Brazil) and used to construct a tissue microarray.

Tissue sampling and study design were approved by the Research Ethics Committee of the Federal University of São Paulo. Informed consent was obtained from all patients or from their legal guardians. Clinical information was collected from patients' medical records.

### RNA isolation and RT-PCR assays

Forty-eight GBM specimens were available for RNA extraction. Total RNA was isolated using the RNeasy Mini Kit (Qiagen), following the manufacturer's instructions. Potentially contaminating DNA was removed by treating with RNase-free DNase (Qiagen). The resulting RNA concentration was measured spectrophotometrically (NanoDrop 1000 Spectrophotometer, Thermo Fischer Scientific) and the quality of the RNAs was checked by electrophoresis on 1% agarose gel.

CTA expression was determined by the reverse transcription- polymerase chain reaction (RT-PCR). One microgram of each RNA sample was subjected to cDNA synthesis using the SuperScript III First-Strand Synthesis System (Invitrogen). The cDNA obtained was diluted 10X before use.

RT-PCR analysis was limited by the availability of specimens: *OIP5* and *XAGE3* were assessed in all 48 samples, *CTCFL* in 46 cases, and *ACTL8* in 37 cases. The mRNA expression of the selected CTA was determined by using previously published oligonucleotide primers (Supplementary Table S1). All PCR reactions were performed as described in the CTDatabase (http://www.cta.lncc.br) (7). Briefly, after an initial denaturation step for 3 minutes at 95°C, the samples were subjected to 35 cycles of denaturation at 95°C for 15 seconds, annealing of 30 seconds (10 cycles at 60°C, 10 cycles at 58°C and 15 cycles at 56°C) and extension of 30 seconds at 72°C, followed by a final extension of 7 minutes at 72°C. The 12.5μl reaction mixtures contained 1.25μL 10x PCR buffer, 0.25μL MgCl_2_ (50mM), 0.125μL dNTPs (10mM), 0.25μL of each primer (10mM), 0.125μL of Platinum Taq DNA Polymerase (5U/μL) (Invitrogen) and 1μL of cDNA.

Testicular tissue was used as a positive control and the β-ACTIN was used as an internal quantity control. Electrophoresis was done by loading 12μL of each sample on 2% agarose gel and visualized by SYBR Safe staining using the EP AlphaImager (Alpha Innotech). Each RT-PCR experiment was done in duplicate using the same cDNA sample. All cases with a detectable band on both replicates were considered positive. The intensities of the PCR products were heterogeneous, and some specimens yielded only faint bands. These were scored positive only if the result was reproducible by a repeated RT-PCR. Cases with very low transcript levels that were not reproducibly positive were regarded as negative.

### Selection of CTA genes

We devised a strategy to select, from all 153 CTA genes described by Hofmann et al. (5), those genes potentially expressed in GBM and absent in normal brain. First, looking for CTA expressed in GBM, publicly available data on CTA expression in GBM held on the Oncomine (http://www.oncomine.org), NExtBio (http://www.nextbio.com) and SAGE Anatomic Viewer (http://cgap.nci.nih.gov/SAGE/AnatomicViewer) databases was mined. Second, the high-throughput expression data (EST, MPSS, CAGE) provided by Hofmann et al (5), the published data of CTA mRNA expression in cell lines (CTDatabase, http://www.cta.lncc.br/index.php), as well as, the data available in the literature, was evaluated. This allowed selection of the most promising CTA genes for subsequent evaluation of their expression in the GBM samples.”

### Tissue Microarray

For tissue microarray (TMA) construction, haematoxylin / eosin sections were analyzed and representative areas of tumors marked on the slides. Tissues corresponding to selected areas were taken from the donor block, using a tissue microarrayer (Beecher Instruments). Each sample was arrayed once with a 0.6-mm diameter core spaced 0.2-mm apart. The sampling consisted of four malignant cores from different areas of the tumor, placed coordinately in the array. Separate samples of normal brain were used as controls. After completion of the array, slides containing serial sections of 4 μm of the TMA block were prepared. Two sections, 40 μm distal from each other, were used for the immunohistochemical study.

### Immunohistochemistry

The expression of CTCFL protein was investigated in astrocytoma samples arranged in a TMA. Rabbit polyclonal antibody against CTCFL protein (1:100 dilution; HPA001472, Sigma) was used as the primary antibody. Four-μm serial sections of the specimens were rehydrated and incubated in 3% aqueous hydrogen peroxide for 30 min to quench endogenous peroxidase activity. Incubation with 1% bovine serum albumin and 5% fetal calf serum in Tris-HCl pH 7.4 was performed for 60 min at room temperature to suppress non-specific binding of subsequent reagents. The sections were then submitted to antigen retrieval with citrate pH 6.0, followed by incubation with primary antibodies. A standard peroxidase-conjugated streptavidin-biotin method was used to detect the staining reaction (LSAB+; DAKO). External positive control tissues included placenta and normal testis samples. A semi-quantitative scoring method was used to evaluate immunohistochemical staining: negative reaction (-): <10% positively stained cells; weakly positive reaction (+): 10% to 25% of cells show positive reaction; moderately positive reaction (++): 25% to 50% of tumor cells with positive reaction; strongly positive reaction (+++): more than 50% of tumor cells with positive reaction. Results of immunohistochemical data were grouped into negative and positive. Tumors with negative and weakly positive immunohistochemical reaction were considered negative, while tumors with moderately positive and strongly positive immunohistochemical reaction were considered positive.

### Cell lines treatment with 5-Aza-dC

A172 and T98G human GBM cell lines were grown in DMEM (Invitrogen) supplemented with 10% fetal bovine serum and 40μL/mL garamycin. Cells were maintained at 37°C in a humid atmosphere containing 5% CO_2_. Cells were counted and plated in 75-cm dishes 1 day prior to treatment, and then the media was removed and replaced with fresh media containing 5μM of 5-aza-2'-deoxycytidine (decitabine, DAC; Sigma) for 3 consecutive days.

### Statistical Analysis

Statistical analysis was performed using MedCalc (ver. 12.3.0). The Chi-square test and Fisher's exact test were used to evaluate the associations between CTA expression and clinical variables, as appropriate. The Kaplan-Meier method was used to estimate overall survival of patients, and differences between groups were compared using the log-rank test. Overall survival (OS) was measured as the time from the date of the surgery for the primary tumor until the date of death from any cause. Multivariate analyses were performed using the Cox proportional hazard model to determine the independent contribution of significant clinical and molecular variables. A stepwise elimination procedure was used for model selection, with a P = 0.2 threshold for elimination. Results were calculated with 95% confidence intervals (CI). For all analyses, a P value was less than 0.05 was considered statistically significant.

## Supplementary Figures and Tables




