# Supplementary material for: Expression of Cancer/Testis Antigens is Correlated with Improved Survival in Glioblastoma
Source: Oncotarget. 2013 Apr 15;4(4):636–46. doi: 10.18632/oncotarget.950 (PMC3720610; doi:10.18632/oncotarget.950)
Supplement: Supplementary file 1 [file oncotarget-04-636-s001.pdf]

Expression of Cancer/Testis Antigens is Correlated with Improved Survival in Glioblastoma- Pereira Freitas

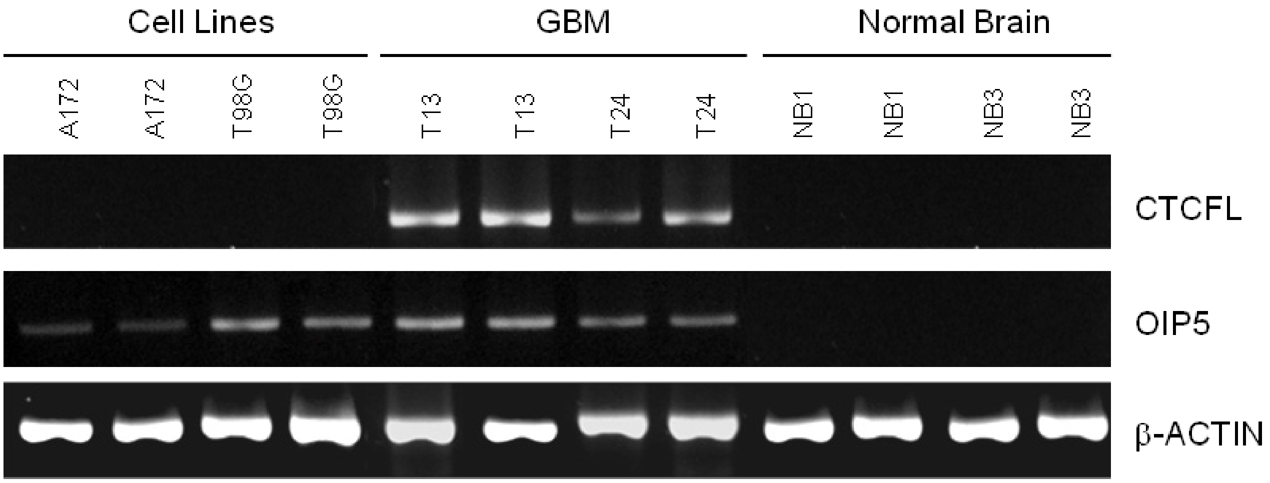

Supplementary Figure S1: Representative samples of typical electrophoresis by RT-PCR of CTCFL and OIP5 from cDNA of glioblastoma multiforme (GBM) cell lines, tumor samples and normal brain tissues on Sybr-safe-stained agarose gels.
