# Supplementary material for: Expression of Cancer/Testis Antigens is Correlated with Improved Survival in Glioblastoma
Source: Oncotarget. 2013 Apr 15;4(4):636–46. doi: 10.18632/oncotarget.950 (PMC3720610; doi:10.18632/oncotarget.950)
Supplement: Supplementary file 2 [file oncotarget-04-636-s002.docx]

EXPRESSION OF CANCER/TESTIS ANTIGENS IS CORRELATED WITH IMPROVED SURVIVAL IN GLIOBLASTOMA - Pereira Freitas

**Supplementary Table S1**: Primers used in the RT-PCR assays

| **Gene** | **Forward 5´-3´** | **Reverse 5´-3´** |
| --- | --- | --- |
| *ACTL8^1^* | GGGTGTGCAGTACCTCTGGT | TGAGGAGATAGGCGGAGAGA |
| *BAGE^1^* | CGGCTTAGAGGACCAGGAG | ACCATCCTGGCTGACACAGT |
| *BRDT^1^* | TCAGAAAGGCACTCAACAG | TTCACTACTTGCTTTAACTGC |
| *CSAG2^1^* | TCCTGGAAAAGGAATTCTACCA | CCGCTATGGCTCATTTTGAT |
| *CTAG1A^2^* | CAGGGCTGAATGGATGCTGCAGA | GCGCCTCTGCCCTGAGGGAGG |
| *CT45^2^* | CTACTTCCCTTCCTCCCATC | GCATGTCCTGTCATAAGCTTC |
| *CTCFL^1^* | CTGCAAGCAGGAACGTCATA | ACTGTTCTCCCTTCGTGGTG |
| *CXORF48^1^* | ATGGCATGATTGATGAGTCG | ATACAACGGATGGGCTTCAC |
| *CXORF61^2^* | TTCCCAACTACCAGCTGAGG | AACCAGCTTGTTTTCCACCA |
| *DDX53^1^* | GGTGCCGATACTCCCACTAT | TTGCTTCAGATTCCCCGTTT |
| *FTHL17^1^* | TGGCCCTGGAGAACTTCTT | ATGGTCTTGACTTGCTCGTG |
| *MAGEA1^1^* | CGGCCGAAGGAACCTGACCCAG | GCTGGAACCCTCACTGGGTTGCC |
| *MAGEA2^1^* | CTCAGGGAGTTGATGACCTTG | GCCTCAAGGCCTTCTTCAG |
| *MAGEB4^4^* | AGGGATACTGCCTCCAGCTC | CAGGAACTGCACTAACATCTTC |
| *MAGEB6^1^* | TGAGCCTGAAAAGTGCTGTC | AGTGGGTGACACTCCCTGAG |
| *NXF2^1^* | CTATTCCCTTCGACCCCA | CTCTTTGGGTGGTTATGTCAC |
| *OIP5^1^* | AGTGGGATACGCAGGTGGT | CTGGAAAGGCAGAAGTGACC |
| *PBK^2^* | TGGCTTTGGTACTGGGGTAA | GCTGCTGGAAAAGGATCTTG |
| *ROPN1^1^* | GGGCAGAGCTAACACCTGAG | TGCTGACATGTGATGCAGAG |
| *SCLO6A1^5^* | TCATATGCCTAGCTCTGTCAAAAG | TCCCGGGTCTGGCATCAATAAAAT |
| *SPANXN1^6^* | TAATGCCCATGGAACAGCCCACTTCAAGC | TAATGCCTCGAGTCTCAGGACTGGTCATTCTCC |
| *SPANXN2^6^* | TAATGCCCATGGAACAGCCGACTTCAAGC | TAATGCCTCGAGCTAGTCCTCCCCACCCTCCTG |
| *SPANXN3^6^* | TAATGCCCATGGAACAGCCAACTTCCAGC | TAATGCCTCGAGCTAATCCTCCCCACTGTCCTG |
| *SPANXN4^6^* | TAATGCCCATGGAAGAGCCAACTTCCAGC | TAATGCCTCGAGCTAATTCTGCCCACCATCCTG |
| *SPINLW1^1^* | AGGACAGACAATGCCAGGAC | GCAGCCACCATAGACAAACA |
| *SPO11^1^* | ACATTTCAGCGGCTCCTAGA | GGTCCCTTTTTGTCAGTGGA |
| *SSX7^7^* | TTTGCAAGGAGACCTAGGGC | GGGGAGTTACTCGTCGTCTTCT |
| *XAGE1^1^* | TCCCAGGAGCCCAGTAATGGAGA | CAGCTTGTCTTCATTTAAACTTGTGGTTGC |
| *XAGE3^1^* | TGAAATATGATTTGGCGAGGA | AAATTGTTCTGATTTTGGCAGA |
| *ZNF165^2^* | GGCCTGGGTACATGAACATT | TTTACCCTGCCTGCAGACTT |

(1) Available at http://www.cta.lncc.br/ (2) Hofmann O, Caballero OL, Stevenson BJ, Chen YT, Cohen T, Chua R, Maher CA, Panji S, Schaefer U, Kruger A, Lehvaslaiho M, Carninci P, Hayashizaki Y, Jongeneel CV, Simpson AJ, Old LJ, Hide W. Genome-wide analysis of cancer/testis gene expression. PNAS December 23, 2008 vol. 105 no. 51 20422-20427. (3) Andrade VC, Vettore AL, Regis Silva MR, Felix RS, Almeida MS, de Carvalho F, Zago MA, Caballero OL, Simpson AJ, Colleoni GW. Frequency and prognostic relevance of cancer testis antigen 45 expression in multiple myeloma. Exp Hematol. 2009 Apr;37(4):446-9. (4) Lurquin C, De Smet C, Brasseur F, Muscatelli F, Martelange V, De Plaen E, Brasseur R, Monaco AP, Boon T. Two members of the human MAGEB gene family located in Xp21.3 are expressed in tumors of various histological origins. Genomics. 1997 Dec 15;46(3):397-408. (5) Lee SY, Williamson B, Caballero OL, Chen YT, Scanlan MJ, Ritter G, Jongeneel CV, Simpson AJ, Old LJ. Identification of the gonad-specific anion transporter SLCO6A1 as a cancer/testis (CT) antigen expressed in human lung cancer. Cancer Immun. 2004 Nov 17;4:13. (6) Kouprina N, Noskov VN, Pavlicek A, Collins NK, Schoppee Bortz PD, Ottolenghi C, Loukinov D, Goldsmith P, Risinger JI, Kim JH, Westbrook VA, Solomon G, Sounders H, Herr JC, Jurka J, Lobanenkov V, Schlessinger D, Larionov V. Evolutionary diversification of SPANX-N sperm protein gene structure and expression. PLoS One. 2007 Apr 4;2(4):e359. (7) Güre AO, Wei IJ, Old LJ, Chen YT. The SSX gene family: characterization of 9 complete genes. Int J Cancer. 2002 Oct 10;101(5):448-53.

**Supplementary Table S2**: Clinical characteristics of GBM patients included in the study (n = 48). One patient was treated in another hospital and lost to follow-up.

| Characteristic | | N | % |
| --- | --- | --- | --- |
| Age | < 50 y.o. | 13 | 27 |
|  | ≥ 50 y.o. | 35 | 73 |
| Extent of surgical ressection | Biopsy | 11 | 23 |
|  | Subtotal | 21 | 44 |
|  | Gross total | 15 | 33 |
| Radiotherapy | Yes | 30 | 64 |
|  | No | 17 | 36 |
| Chemotherapy | Yes | 20 | 43 |
|  | No | 27 | 57 |
| KPS score | ≤70 | 26 | 55 |
|  | >70 | 21 | 45 |

**Supplementary Table S3:** Relationship among genetic variables and baseline characteristics

|  | ***ACTL8* (+)** | ***ACTL8* (-)** | ***CTCFL* (+)** | ***CTCFL* (-)** | ***OIP5* (+)** | ***OIP5* (-)** | ***XAGE3* (+)** | ***XAGE3* (-)** | **3-4 CTA** | **0-2 CTA** |
| --- | --- | --- | --- | --- | --- | --- | --- | --- | --- | --- |
| **Age** |  | |  | |  | |  | |  | |
| <50 years | 7 | 3 | 4 | 8 | 7 | 6 | 7 | 6 | 6 | 4 |
| ≥50 years | 14 | 13 | 3 | 31 | 19 | 16 | 16 | 19 | 22 | 4 |
| *p* | 0.461 | | 0.064 | | 0.765 | | 0.860 | | 0.179 | |
| **KPS** |  | |  | |  | |  | |  | |
| >70 | 6 | 8 | 4 | 17 | 14 | 7 | 12 | 9 | 4 | 10 |
| ≤70 | 15 | 8 | 3 | 21 | 11 | 15 | 10 | 16 | 4 | 18 |
| *p* | 0.322 | | 0.688 | | 0.170 | | 0.326 | | 0.683 | |
| **Surgery** |  | |  | |  | |  | |  | |
| Ressection | 15 | 14 | 6 | 29 | 21 | 16 | 18 | 19 | 7 | 21 |
| Biopsy | 6 | 2 | 1 | 9 | 4 | 6 | 4 | 6 | 1 | 7 |
| *p* | 0.422 | | 1 | | 0.479 | | 0.729 | | 0.651 | |
| **Radiotherapy** |  | |  | |  | |  | |  | |
| Yes | 13 | 10 | 6 | 24 | 18 | 12 | 17 | 13 | 7 | 16 |
| No | 8 | 6 | 1 | 16 | 7 | 10 | 5 | 12 | 1 | 12 |
| *p* | 0.760 | | 0.395 | | 0.348 | | 0.676 | | 0.212 | |
| **Chemotherapy** |  | |  | |  | |  | |  | |
| Yes | 9 | 7 | 4 | 16 | 13 | 7 | 9 | 11 | 4 | 12 |
| No | 12 | 9 | 3 | 22 | 12 | 15 | 13 | 14 | 4 | 16 |
| *p* | 0.779 | | 0.682 | | 0.271 | | 0.934 | | 1 | |
